# The patterns of vascular plant discoveries in China

**DOI:** 10.1002/ece3.7971

**Published:** 2021-08-24

**Authors:** Muyang Lu, Lianming Gao, Hongtao Li, Fangliang He

**Affiliations:** ^1^ ECNU‐Alberta Joint Lab for Biodiversity Study Tiantong National Station for Forest Ecosystem Research East China Normal University Shanghai China; ^2^ Ecology and Evolutionary Biology Yale University New Haven CT USA; ^3^ CAS Key Laboratory for Plant Diversity and Biogeography of East Asia Kunming Institute of Botany Chinese Academy of Sciences Kunming China; ^4^ Germplasm Bank of Wild Species in Southwest China Kunming Institute of Botany Chinese Academy of Sciences Kunming China; ^5^ Department of Renewable Resources University of Alberta Edmonton AB Canada

**Keywords:** biodiversity hot spots, botanical discovery, conservation prioritization, Flora of China, species accumulation curve, survival analysis, taxonomic efforts

## Abstract

**Aim:**

(1) To understand geographic patterns of species discovery by examining the effect of growth form, range size, and geographic distribution on discovery probability of vascular plant species in China; (2) to find out which taxa harbor the largest number of undiscovered species and where those species locate; and (3) to find out the determinants of province‐level mean discovery time and inventory completeness.

**Location:**

China.

**Methods:**

We compiled the discovery time and province‐level geographic distributions of ~31,000 vascular plant species described between 1753 and 2013 from *Flora of China*. We used a Cox proportional hazard model to determine the biological and geographic correlates of discovery probability. Accumulation curves of species discoveries were fitted by a logistic discovery model to estimate inventory completeness of different growth forms and of different provinces. We then used linear regression to identify the determinants of mean discovery time and beta regression to identify the determinants of inventory completeness.

**Results:**

We found that species with larger range size and distributed in northeastern part of China have a higher discovery probability. Coastal species were discovered earlier than inland species. Trees and shrubs of seed plants have the highest discovery probability while ferns have the lowest discovery probability. Herbs have the largest number of undiscovered species in China. Most undiscovered species will be found in southwest China, where three global biodiversity hot spots locate. Spatial patterns of mean discovery time and inventory completeness are mainly driven by the total number of species, human population density in an area, and latitude and longitude of a province.

**Main Conclusions:**

Socioeconomic factors primarily determine the discovery patterns of vascular plants in China. Undiscovered species are most likely to be narrow‐ranged, inconspicuous endemic species such as herbs and ferns, which are prone to extinctions and locate in biodiversity hot spots in southwestern China.

## Graphical abstract

Botanical discoveries in China were shaped by historical events and socioeconomic factors, and driven by plant species' range size and growth forms. We highlight that ferns and herbs are the most underdiscovered groups, and future discoveries are most likely to be made in the southwestern China.

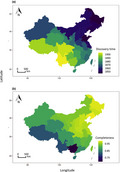

## 1 INTRODUCTION

Despite more than 260 years’ discovery of species since Linnaeus, our knowledge about the biological diversity is still far from complete (Bini et al., 2006; Hortal et al., 2015). According to a previous estimate, fewer than 20% of species on Earth have been discovered so far (Mora et al., 2011). With the ongoing biodiversity crisis, this lack of knowledge (known as the Linnean Shortfall; Brown & Lomolino, 1998) has become a major obstacle to biodiversity conservation as many species could go extinct without ever being known to science (Costello et al., 2013; Humphreys et al., 2019).

Early species discoveries are often biased toward large‐sized, charismatic species with widespread geographic distributions (Essl et al., 2013; Ferretti et al., 2008; Gibbons et al., 2005; Randhawa et al., 2015; Stork et al., 2008, 2015). For instance, positive correlations between species discovery probability and body size have been found in a variety of taxa including insects (Gaston & Hudson, 1994), birds (Blackburn & Gaston, 1995), mammals (Collen et al., 2004; Medellín & Soberón, 1999; Paxton, 1998), fishes (Zapata & Robertson, 2007), reptiles, amphibians (Moura & Jetz, 2021; Reed & Roback, 2002), and marine holozooplanktons (Gibbons et al., 2005). More recently discovered species are often of greater conservation interest because they are more likely to be narrow‐ranged and rare and thus more prone to extinctions (Bebber et al., 2007, 2010; Diniz‐Filho et al., 2005; Tedesco et al., 2014; Xu et al., 2019).

In addition to biological factors, species discovery is also influenced by human factors such as taxonomic effort, technology innovations, and socioeconomic events. For example, it is known that species discovery rates for many taxa dropped during the two World Wars and peaked in the 1990s with the emergence of molecular techniques (Bebber et al., 2007; Gaston, 1995; Joppa et al., 2011; Lu & He, 2017). Geographically speaking, Europe and North America have the most complete species inventories due to their long histories of exploration and larger taxonomic workforce (Essl et al., 2013; Gaston, 1995), while species discovery in less explored continents such as South America and Africa was affected by colonization histories and indigenous knowledge (Ballesteros‐Mejia et al., 2013; Diniz‐Filho et al., 2005; Gaston, 1995; Moura & Jetz, 2021; Rosenberg et al., 2013). As a result, biodiversity hot spots, most of which are located in developing countries (Myers et al., 2000), often harbor the largest number of undiscovered species (Giam et al., 2012; Joppa et al., 2011). This imposes a more serious challenge for biodiversity conservation in developing regions where economic growth is often achieved at the expense of environmental degradation (He, 2009).

China harbors nearly one tenth of the plant species on Earth (Joppa, Roberts, & Pimm, 2011; Lu & He, 2017). However, its rapid economic growth over the past three decades has resulted in the colossal loss of millions of hectares of natural habitats (He et al., 2014; He, 2009). The sustainable development of China depends on balancing economic growth and preservation of natural habitats. Knowing where undiscovered species may locate is necessary for making decision on habitat protection and conservation management.

The interest for cataloging species in China has long predated the invention of Linnaeus’ binomial nomenclature. But in light of modern taxonomy, much credit should be given to Western naturalists who diligently collected specimens and described species since the first arrival of Jesuits in China in the 16th century, as reflected by the fact that nearly 70% of the type specimens of Chinese plants are kept in herbaria in Europe and North America (Chen, 1994). Due to logistic constraints and political instability, most naturalists in the 18th and 19th centuries made their botanical collections in the coastal areas of China (Bretschneider, 1898; Fan, 2004), which likely had affected the patterns of collection records. There are more than 31,000 vascular plant species documented in China, but this inventory is not complete and many new species, estimated to be nearly 15% of them, still await discovery (Lu & He, 2017). Knowing the traits of those inconspicuous species and where they may locate is important for future taxonomic efforts. Therefore, the objectives of this study are (a) to model and map geographic variation in botanical discovery of vascular plant species in China, (b) to find out which growth form contains the largest number of undiscovered species and where these species most likely are, and (c) to quantify what factors (e.g., human population density and species richness) may influence the spatial distribution of plant discoveries in China. This study will contribute to understanding the pattern of species discoveries and their underlying factors, which should be of significance to botanical discoveries of regions beyond China. The identification of taxonomic and geographic gaps of undiscovered species will facilitate prioritizing our limited taxonomic and conservational efforts in future.

## 2 METHODS

### 2.1 Data

Data including species names, discovery time, province‐level biogeographic distributions, and genus‐level growth forms were compiled from Flora of China (FOC http://efloras.org; compiled data available on Dryad: https://doi.org/10.5061/dryad.4b8gthtd1), which has a total number of 31,093 species for analysis after cleaning. We treated discovery time as the time a species was first described in a scientific publication. If the species was first described in a synonym, the publication time of the synonym was used. When estimating the number of undiscovered species, data after 2000 were excluded as a routine to avoid the effect of delayed entrance of newly discovered species (Costello & Wilson, 2011; Costello et al., 2012). Human population densities at the province‐level were obtained from *2010 Population Census of The People's Republic of China* (http://www.stats.gov.cn/) and *Monthly Bulletin of Interior Statistics* (http://sowf.moi.gov.tw/stat/month/elist.htm). Province areas were obtained from *National Fundamental Geographic Information System of China* (http://nfgis.nsdi.gov.cn/nfgis/). There are in total 28 provinces after merging municipalities such as Beijing and Shanghai to adjacent provinces. Range size, maximum/minimum latitude, maximum/minimum longitude, and whether or not a species is distributed in coastal areas (provinces adjacent to the sea) were obtained from province‐level distributions. Genus‐level growth forms were categorized as ferns, herbs, shrubs/trees, and vines/lianas. Shrubs and trees were categorized as one group because many species have both shrubs and trees as growth forms. Vines and lianas include herbaceous vines, woody lianas, and all other plants with climbing forms. When a genus has several different growth forms, we used the primary growth form (which has the largest number of species within the genus) as the genus‐level growth form. We used Turkey's range test for the multiple comparison of mean discovery time among different growth forms. Range size, maximum/minimum latitude, and maximum/minimum longitude were standardized to [0, 1] in order to calculate the effect size on discovery probability. Range size was log‐transformed before standardization. Correlations between explanatory variables were checked prior to analysis. No collinearity was found among explanatory variables (maximum VIF = 5.9 < 10).

### 2.2 Cox proportional hazard model

We first modeled the discovery time using survival analysis (Bebber et al., 2010; Essl et al., 2013). Survival analysis is used to analyze time‐to‐event data where the response is a duration of time. In this study, the event is the discovery of a species. The discovery time is then calculated as the publication year minus 1753 (the time Linnean nomenclature was established). Because only discovered species could be recorded, there are no censored data in our study. In this case, the empirical survival curve is just the inverse of the accumulation curve (Essl et al., 2013; Steyskal, 1965). Cox proportional hazard (Cox PH) model was used to model the instantaneous discovery probability which is a conditional probability that a species will be seen in the next step of time (*t *+ Δ*t*) if it remains unseen up to time *t*. This probability is expressed as a hazard function, *h*(*t*), given as

$$h(t)=\lim_{\Delta t\to 0}\frac{\mathrm{Pr}[(t\leq T<t+\Delta t)|T\geq t]}{\Delta t}.$$

The Cox PH regression model is:

$$h_{i}(t)=h_{0}(t)\exp(\beta X),$$

where matrix *β*X is the linear component of the model (i.e., coefficients and predicting variables). *h_i_* (*t*) is the hazard function for species *i* (i.e., the “risk” to be discovered), and *h*_0_(*t*) is the baseline hazard function. Compared with accelerated failure time model (AFT), Cox model is equivalent to a semi‐parametric model, which makes no assumption about the underlying distribution of survival time (i.e., discovery time), and is more appropriate for our data because botanical discovery is highly influenced by historical events (Lu & He, 2017), and the probability distribution of discovery time is unknown. When multiple species were discovered in the same year, Efron approximation was used to break ties in discovery time (Hertz‐Picciotto & Rockhill, 1997). The proportional hazard assumptions were examined by plotting the scaled Schoenfeld residuals, denoted as *β*(*t*), against time. A horizontal trend of *β*(*t*) implies that the time‐independent coefficient assumption is met. We used the range size, genus‐level growth form, maximum/minimum latitude, maximum/minimum longitude, and coastal distribution of a species as predictors for the Cox PH model. We used step selection to choose the “best” model based on the minimum AIC value (Burnham & Anderson, 2004). Because all variables were significant, we included them all in Table 1. Concordance statistic (*C* statistic) was used to show the discriminative ability of the model. It is equivalent to the area under the receiver operating characteristic curve (AUC) in logistic regression, with the value of 0.5 indicating no discrimination power and the value of 1 indicating perfect discrimination (Hanley & McNeil, 1982). We also presented the fitted survival curves for different treatments (i.e., inland vs. coastal distributed species, and species with different growth forms) using strata models (each treatment has a different baseline function *h*_0_(*t*)). Effect sizes are regression coefficients of the standardized predicting variables. All survival analyses were conducted using package “survival” (Therneau, 2020) in R version 4.0.4 (R Core Team, 2020).

**TABLE 1:** Cox proportional hazard model as a function of biological and geographic predictors for ~31,000 vascular plant species from China

	Effect size	Lower 95%	Upper 95%	Standard error	*p*‐Value
Growth form.fern	−0.82	−0.87	−0.76	0.03	<.001
Growth form.herb	−0.13	−0.16	−0.10	0.01	<.001
Growth form.vine.liana	−0.13	−0.19	−0.07	0.03	<.001
Range size	0.51	0.39	0.63	0.06	<.001
Coast	0.13	0.09	0.16	0.02	<.001
Maximum longitude	0.90	0.80	0.99	0.05	<.001
Minimum longitude	−0.46	−0.56	−0.36	0.05	<.001
Maximum latitude	0.67	0.57	0.78	0.07	<.001
Minimum latitude	−0.73	−0.82	−0.64	0.04	<.001

Note

Growth form was categorical data and tree/shrub was treated as the baseline category. *N* = 30,944. Concordance = 0.653 (*SE* = 0.002).

### 2.3 Estimating species richness

We used a modified logistic species discovery model (Lu & He, 2017) to estimate species richness for different growth forms and for each province:

$$\Delta S_{t}=(a+bS_{t})(S_{\text{tot}}‐S_{t})+\varepsilon_{t},$$

where Δ*S_t_* is the number of species discovered per time interval (5 years in this study), *S*_tot_ is the total number of species in a region, *S_t_* is the accumulative number of species discovered up to time *t* (=0, 5, 10, 15, 20,… years), *a* and *b* are fitting parameters, and *ε_t_* is the error term. Our goal was to estimate *S*_tot_. The model was fitted by generalized nonlinear least‐square regression with R package “nlme” (Pinheiro et al., 2020). Note that the logistic species discovery model is different from the logistic regression model. The “logistic” part of the discovery model derives from the logistic shape of species discovery curve. The logistic species discovery model provides only conservative estimates in certain cases (Bebber, Marriott, et al., 2007; Essl et al., 2013; Lu & He, 2017). Inventory completeness was calculated as the ratio of the number of discovered species to the estimated total number of species in a province or in a growth form.

### 2.4 Spatial patterns of discovery time and inventory completeness

We conducted a spatial analysis to examine the mean discovery time (i.e., the average number of years taken to discover a species in an area) of a province as a function of human population density, total number of species, whether a province is on the coast, and province area using ordinary linear regression. We expect that mean discovery time is negatively correlated with coastal distribution and population density because these areas are more accessible for discovery (Diniz‐Filho et al., 2005). We also expect that the mean discovery time is positively correlated with species richness in a province because it takes longer to discover more species. The spatial autocorrelation of mean discovery time at the province‐level was examined by Moran's *I*. The neighborhood structure of provincial polygons is defined by contiguity (only polygons with shared borders are counted as neighbors). We proceeded with ordinary linear regression after no spatial autocorrelation was detected in the residuals of the model (Figure S1). To further account for differences in range size and species richness among provinces, we also calculated the weighted standardized mean discovery time for each province (using the inverse of species’ range size as weight to downplay the influence of widespread species) using a null model where the discovery times of all species in China were randomly shuffled 1,000 times while fixing the province‐level occurrence pattern (results in Table S2). The standardized mean discovery time of a province was calculated as the observed value subtracted by the mean and divided by the standard deviation obtained from the 1,000 random shuffles (Moura et al., 2018). Positive value (above 1.96) indicates discovery later than expected, and negative value (below −1.96) indicates discovery earlier than expected.

We used beta regression to model province‐level inventory completeness with the same set of covariates as modeling mean discovery time (i.e., population density, total number of species, whether a coastal province or not, and province area) using R package “betareg” (Cribari‐Neto & Zeileis, 2010). Beta regression was used because the response variable is proportional data (not binary data), ranging from 0 to 1. The regression is flexible to accommodate the shape of the distribution (symmetric or skewed). For both spatial analyses of mean discovery time and inventory completeness, we also used step selection to choose the “best” model based on the minimum AIC value (Burnham & Anderson, 2004).

## 3 RESULTS

### 3.1 Cox proportional hazard model for species discovery probability

**FIGURE 1:**
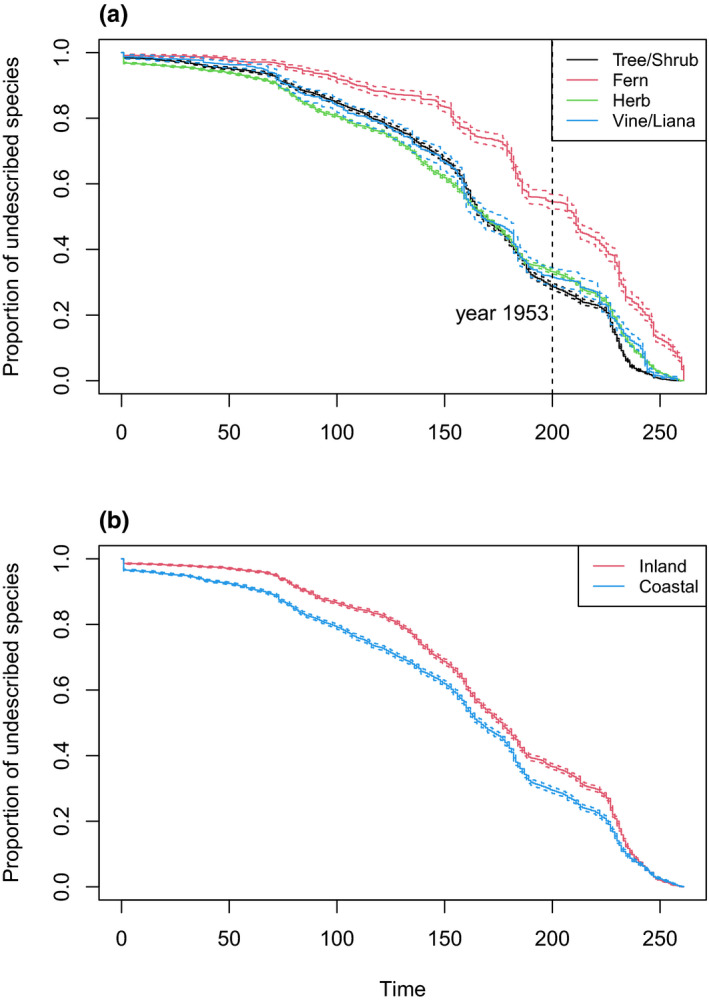
Fitted survival curves of the Cox proportional hazard models (a) stratified on variable “growth form” and (b) “coast.” Dashed lines show 95% confidence intervals

Ferns had the lowest discovery probability; trees and shrubs had the highest discovery probability among all groups (Table 1). For example, in the year 1953 (200 years after botanical discovery in China), ferns remained the least discovered group and trees and shrubs the most discovered group (Figure 1a). Coastal species were discovered earlier than inland species (Figure 1b). The discovery probability of a species increases with its range size, maximum latitude, and longitude and decreases with minimum latitude and longitude. The effect sizes of maximum latitude and longitude and minimum latitude and longitude were larger than the effect sizes of range size, coastal distribution, and growth form, suggesting the importance of geographic locations to discovery probability (Table 1).

### 3.2 Estimating species richness

**FIGURE 2:**
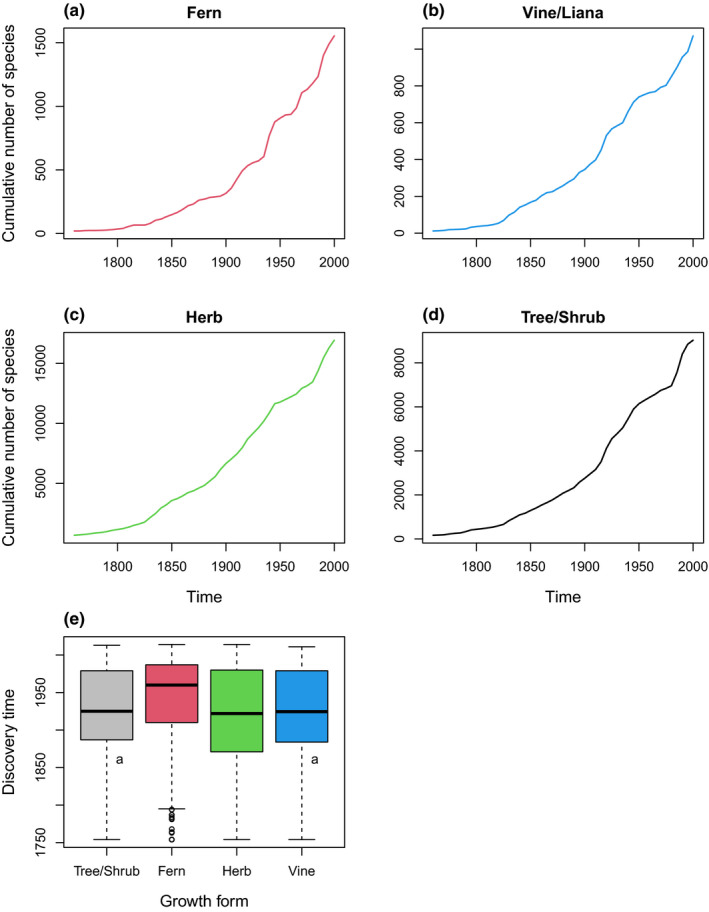
(a–d) Species accumulation curves for four growth forms (based on 5‐year interval data). (e) Boxplot for discovery time of the four growth forms. “a” labels the groups with no significant difference in Turkey's range test

The logistic model estimated that the inventory completeness of ferns is 0.62 (Table 2), suggesting that there remain a considerable number of fern species to be discovered in future. This is consistent with the steep accumulation curve in recent years (Figure 2a). For seed plants, the estimated inventory completeness is 0.73 for herbs, 0.75 for shrubs and trees, and 0.68 for vines/lianas (Table 2). Herbs harbor the largest number of undiscovered species. The low inventory completeness of ferns is consistent with the fact that it took on average the longest time to discover a fern species (*p* < .05 for all pairs except between tree/shrub and vine; Figure 2e).

**TABLE 2:** Estimated species richness for different growth forms based on the logistic discovery model

	Number of discovered species	Estimated total number of species	Lower 95% bound	Upper 95% bound	Completeness (lower 95%‐)
Fern	1,921	2,712	749	4,676	0.622 (0.41‐)
Vine/Liana	1,202	1,710	856	2,563	0.685 (0.47‐)
Herb	18,370	23,867	16,982	30,752	0.737 (0.59‐)
Tree/Shrub	9,451	12,998	8,855	15,741	0.754 (0.60‐)

### 3.3 Spatial patterns of mean discovery time and inventory completeness

**FIGURE 3:**
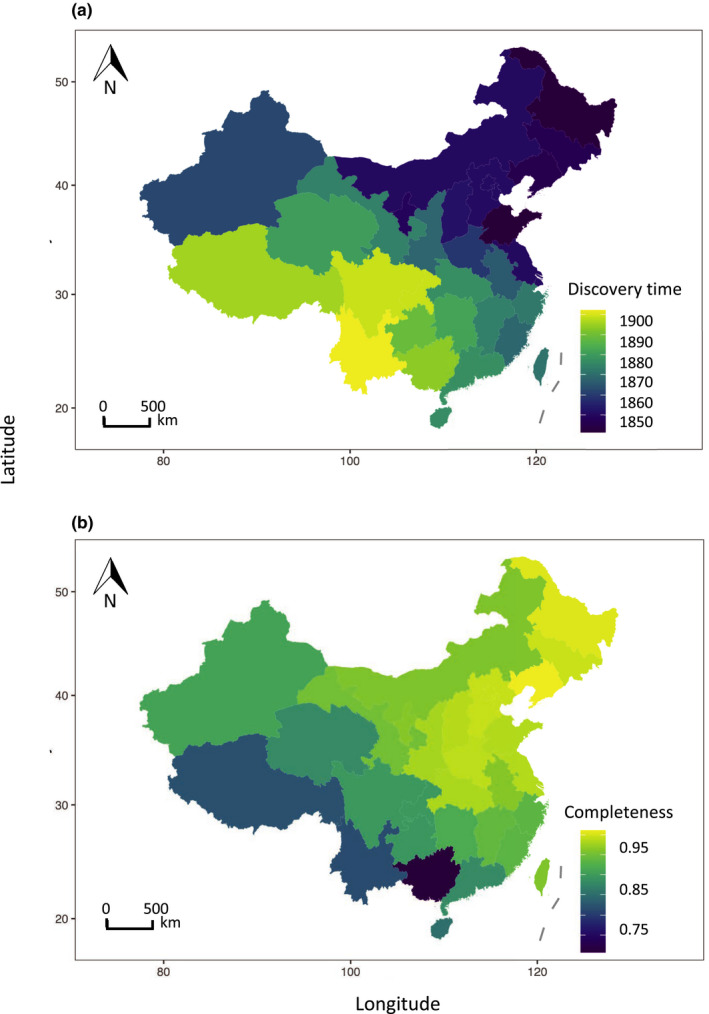
(a) Distribution of mean province‐level species discovery time in China. (b) Distribution of species inventory completeness. Mollweide projection is used for mapping

**FIGURE 4:**
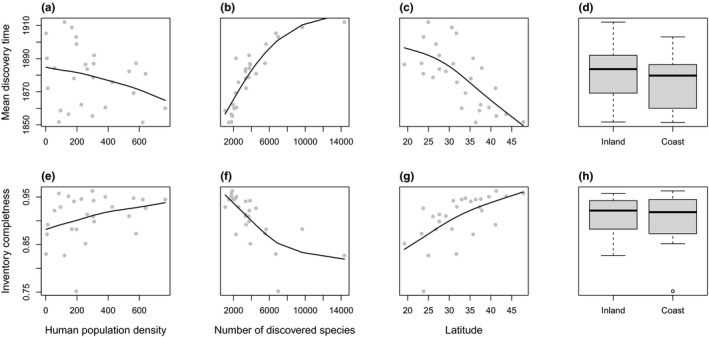
(a–c) Mean species discovery time against human population density, number of discovered species, and latitude. (d) Boxplot of mean discovery time for inland and coastal provinces. (e–g) Species inventory completeness against human population density, number of discovered species, and latitude. (h) Boxplot of inventory completeness for inland and coastal provinces. Solid lines show the fitted smooth spline curves

The mean discovery time increases from northeast toward southwest (Figure 3a). Human population density is positively correlated with mean discovery time (Figure 4a) but it was removed from the multiple regression after model selection. The best model explains 89% of the total variation of mean discovery time (Table 3) which is positively correlated with total number of species (Figure 4b), negatively correlated with latitude (Figure 4c), and is shorter in coastal provinces than in inland provinces (Figure 4d). When accounted for differences in range size and species richness, the standardized mean discovery time is positively correlated with the total number of species while negatively correlated with province area and longitude (Table S2).

**TABLE 3:** Linear regression of province‐level mean discovery time

	Coefficient	Lower 95% CI	Upper 95% CI	Standard error	*p*‐Value
Intercept	1,902.22	1,889.93	1,914.52	6.27	<.001
Number of species	35.79	20.43	51.14	7.83	<.001
Coast	−7.55	−13.79	−1.30	3.18	.012
Mean longitude	−17.98	−30.28	−5.68	6.27	.004
Mean latitude	−37.30	−50.41	−24.19	6.69	<.001

Note

Model with the minimum AIC was selected by step selection. Predictors standardized between 0 and 1. Adjusted *R* ^2^ = .87.

We estimate that in 18 of the 28 (64.3%) provinces, plant species discoveries are more than 90% complete. Provinces with the largest proportion of undiscovered species are in southwest China (75.1% in Guangxi province and 82.7% in Yunnan province; Figure 3b). Beta regression explains 85% of total variation in inventory completeness, which is positively correlated with human population density, the latitude of a province, and negatively correlated with area (Table 4).

**TABLE 4:** Beta regression of province‐level inventory completeness

	Coefficient	Lower 95% CI	Upper 95% CI	Standard error	*P*‐value
Intercept	1.29	1.04	1.55	0.13	<.001
Human population density	0.78	0.38	1.19	0.21	<.001
Area	−0.85	−1.28	−0.43	0.22	<.001
Latitude	2.01	1.63	2.40	0.19	<.001

Note

Model with the minimum AIC was selected by step selection. Predictors standardized between 0 and 1. Pseudo *R* ^2^ = .85.

## 4 DISCUSSION

Today's knowledge about biodiversity is the result of arduous quest of generations of naturalists for species discoveries. Although the nomenclatures of species are universally binomial, the stories behind their discoveries are not and many of them are as colorful as the species that were discovered (Kilpatrick, 2014). The rich information provided by discovery history is especially valuable for filling the knowledge gap in biodiversity research (Meyer et al., 2015, 2016) because it provides guidance about when and where future discoveries are going to be made and what traits influence future discoveries (Collen et al., 2004; Diniz‐Filho et al., 2005; Moura & Jetz, 2021). Therefore, knowledge on species discovery is of great value for species conservation if we strive to describe all species before they go extinct (Costello et al., 2013; Essl et al., 2013; Joppa, Roberts, Myers, et al., 2011; Tedesco et al., 2014). In this study, we compiled data on vascular plant species discovered over 260 years in China for understanding the geographic variation of discovery time and the completeness of botanical inventory of the country.

Our analysis shows that vascular plant species with a larger range size were discovered earlier in China, consistent with previous findings that widespread species were more easily discovered in the history (Bebber, Harris, et al., 2007; Essl et al., 2013). Tree and shrub species of seed plants have the highest discovery probability while fern species have the lowest discovery probability. Herb and vine/liana species of seed plants all have similar discovery probabilities (Table 1). Species distributed on the coast have a higher discovery probability than inland species even when geographic information such as the latitudinal range of a species is included in the model (Table 1), which is also shown by the result that province‐level mean discovery time is negatively correlated with coastal distribution (Table 3). This is likely because coastal areas in China were most economically developed and much more accessible to Western naturalists since the Opium War (Bretschneider, 1898; Fan, 2004).

Our results indicate that fern is likely the most underdiscovered plant taxon in China because the species discovery curve for fern shows little sign of level‐off (Figure 2a, Table 2). Herbs have the largest number of undiscovered species (Table 2) and the second lowest discovery probability estimated from the Cox proportional hazard model (Table 1). Higher discovery probability usually leads to higher inventory completeness, which is shown by the concordance between ranks of discovery probability and ranks of inventory completeness among groups in our results (Tables 1 and 2). Although the inventory completeness estimated from this study varies among the growth forms, their differences are relatively small (~2% between herb and tree/shrub). Herbs have the largest number of undiscovered species likely because the total number of herb species is larger than that of any other growth form of seed plants in China. We suspect that the effect of growth form on discovery probability and inventory completeness at least partially reflects the difference in the availability of taxonomic expertise, especially for ferns. The description of fern species started relatively late in China (~1920s; Chen, 1994) compared with other groups likely because of the difficulty in distinguishing subtle morphological characters, the lack of taxonomic expertise, and more labile species concept at that time (Christenhusz & Chase, 2014). Given that herbs also contain the largest number of undiscovered species and that many specimens of undescribed species have already been preserved in herbaria or museums (Bebber et al., 2010; Fontaine et al., 2012; Guedes et al., 2020), our study suggests that the lack of taxonomic expertise might be the primary limiting factor of discovering new species in China, which resonates with the call to address the challenge of “taxonomic impediments” (Bebber et al., 2014; Ebach et al., 2011; Ma, 2014).

The spatial pattern of mean species discovery time is driven by species diversity in an area and geographic locations (Table 3 and Table S2), while inventory completeness is driven by human population density (Table 4). Although human history did affect the spatial patterns of species discovery (Ballesteros‐Mejia et al., 2013; Diniz‐Filho et al., 2005; Rosenberg et al., 2013), geographic sampling bias does not change the prioritization of the current conservation efforts because the total number of species and number of discovered species are highly correlated (Giam et al., 2012; Joppa, Roberts, Myers, et al., 2011; Figure S4). We expect that new discoveries in future are most likely to be made in interior southwestern provinces with high species richness such as Xizang, Guangxi, and Yunnan.

The spatial pattern of species inventory completeness at the province level is at odds with a previous study showing that at the county level eastern China has lower inventory completeness (Yang, Ma, & Kreft, 2013, 2014). Yang et al. measured county‐level inventory completeness with the slope of sample‐based accumulation curves using specimen collections. However, the slope of species accumulation curve is not a genuine measure of inventory completeness. Rather, it measures the variation in species composition in the samples used to construct the species–accumulation curve (Thompson & Withers, 2003). Therefore, their assessment of inventory completeness could be biased by a sampling strategy that aims at collecting as many novel species as possible for a given amount of samples (Chen, 1994). Another possible reason for this discrepancy is that inventory completeness is scale‐dependent. In a hypothetical scenario, even if the inventory completeness at the county level is on average 90%, the inventory completeness at the province level could still be lower than 90% if most of the recorded species at the county level are common species. While Yang et al. (2014) argued that more efforts should be devoted to increasing botanical collections in eastern densely populated areas, our study does not support this advocacy. Instead, we suggest that future botanical collection efforts should be more allotted to the provinces of southwest China where there is high species diversity and the botanical inventory is least complete especially in the face of rapid habitat loss in recent years (He et al., 2014). Recent findings support our conclusion by showing that the majority of newly discovered species (73%) in China after the completion of *Flora of China* in 2013 came from the global biodiversity hot spots of Indo‐Burma and mountains of southwest China (Cai et al., 2019); Yunnan, Guangxi, Sichuan, Xizang and Taiwan are provinces where most new species were discovered during 2000–2019 (Du et al., 2020).

We identified two possible limitations in this study. The first one is that species discovery time is not the time when the species was first collected in the field but the time when the species was first described in a scientific publication. This could lead to inflating discovery probability for widespread species if their type specimens were collected outside of China. To address this problem, we reran our Cox proportional hazard model using species only endemic to China (12,917 species). The results show that the key drivers of discovery probability are mostly consistent but with some noticeable differences (Table S1): The effect size of coastal distribution became negative because the majority of these endemic species were discovered after 1900 (Figure S2a), which removed the effect of early discoveries made by Western naturalists. For the province‐level analysis, excluding nonendemic species in China does not significantly change the spatial pattern of mean discovery time (Figure S3a). Human population density became negatively correlated with the mean discovery time of endemic species; area became positively correlated with mean discovery time; and the effect of coastal distribution and number of species are no longer significant (Table S3). The patterns of the standardized mean discovery time for the endemic species are consistent with the patterns for all species in China (Tables S2 and S4). For province‐level inventory completeness, the effects of predictors are mostly consistent with the model that includes all species except that coastal distribution (Table S5).

The second limitation is that our data do not distinguish the discovery of a new species from a species resurrected or revalidated from a known synonym. As data including a full list of all synonyms at each time step are not available, we are not able to model the transition rate from synonyms to valid names (Alroy, 2002).

In summary, our study shows that most underdiscovered vascular plant species in China are ferns and herbs of seed plants, which are mostly narrowly distributed endemic species in the southwest biodiversity hot spots of China. Given the “taxonomic impediments” we are facing (Ma, 2014), more resources should be channeled to the recruitments and training of taxonomic expertise in these two particular groups. There is an urgency of cataloging undiscovered species in southwest mountainous areas for future conservation designs and botanical study.

## CONFLICT OF INTEREST

None declared.

## AUTHOR CONTRIBUTION

**Muyang Lu:** Conceptualization (lead); Data curation (equal); Formal analysis (lead); Investigation (lead); Methodology (lead); Project administration (lead); Writing‐original draft (lead). **Lianming Gao:** Resources (equal); Validation (supporting); Writing‐review & editing (supporting). **Hongtao Li:** Data curation (equal); Resources (equal); Writing‐review & editing (supporting). **Fangliang He:** Conceptualization (supporting); Funding acquisition (lead); Methodology (supporting); Project administration (supporting); Resources (equal); Supervision (lead); Validation (supporting); Visualization (supporting); Writing‐review & editing (equal).

## Supporting information

Appendix S1

## Data Availability

Data used in this study are stored in Dryad (https://doi.org/10.5061/dryad.4b8gthtd1).
