# Supplementary material for: The patterns of vascular plant discoveries in China
Source: Ecol Evol. 2021 Aug 24;11(18):12378–88. doi: 10.1002/ece3.7971 (PMC8462150; doi:10.1002/ece3.7971)
Supplement: Supplementary file 1 — Appendix S1 [file ECE3-11-12378-s001.docx]

Appendix 1: **The patterns of vascular plant discoveries in China**

Table S1. Cox proportional hazard model with all variables for endemic species in China (growth forms were coded as a categorical variable in which tree/shrub was used as the baseline variable).

|  | Effect size | Lower 95% CI | Upper 95% CI | Standard error | *P*-value |
| --- | --- | --- | --- | --- | --- |
| Growth form.fern | -0.83 | -0.91 | -0.76 | 0.04 | <0.001 |
| Growth form.herb | -0.08 | -0.12 | -0.04 | 0.02 | <0.001 |
| Growth form.vine.liana | -0.23 | -0.32 | -0.13 | 0.05 | <0.001 |
| Range size | 1.21 | 1.01 | 1.41 | 0.10 | <0.001 |
| Coast | -0.12 | -0.17 | -0.06 | 0.03 | <0.001 |
| Maximum longitude | 1.30 | 1.11 | 1.50 | 0.11 | <0.001 |
| Minimum longitude | 0.09 | -0.09 | 0.28 | 0.08 | 0.32 |
| Maximum latitude | 0.30 | 0.096 | 0.51 | 0.99 | <0.004 |
| Minimum latitude | -0.70 | -0.87 | -0.54 | 0.94 | <0.001 |
| N = 12917  Concordance = 0.634 (se = 0.003) | | | | | |

Table S2. Linear regression of standardized mean discovery time for all species.

|  | Coefficient | Lower 95% CI | Upper 95% CI | Standard Error | *P*-value |
| --- | --- | --- | --- | --- | --- |
| Intercept | 10.13 | -0.03 | 20.3 | 5.18 | 0.06 |
| Number of species | 30.62 | 20.54 | 40.70 | 5.14 | <0.001 |
| Area | -37.08 | -47.96 | -26.19 | 5.56 | <0.001 |
| Mean longitude | -21.24 | -33.22 | -9.25 | 6.11 | 0.005 |
| Adjusted *R*^2^: 0.79 | | | | | |

Table S3. Linear regression of mean discovery time for endemic species.

|  | Coefficient | Lower 95% CI | Upper 95% CI | Standard Error | *P*-value |
| --- | --- | --- | --- | --- | --- |
| Intercept | 1944.21 | 1939.11 | 1949.31 | 2.60 | <0.001 |
| Human population density | -18.87 | -25.84 | -11.89 | 3.56 | <0.001 |
| Area | 24.79 | 16.57 | 33.00 | 4.19 | <0.001 |
| Mean latitude | -51.01 | -57.88 | -44.32 | 3.46 | <0.001 |
| Adjusted *R*^2^: 0.90 | | | | | |

Table S4. Linear regression of standardized mean discovery time for endemic species.

|  | Coefficient | Lower 95% CI | Upper 95% CI | Standard Error | *P*-value |
| --- | --- | --- | --- | --- | --- |
| Intercept | 8.44 | -0.99 | 17.86 | 4.81 | 0.09 |
| Number of species | 29.22 | 20.86 | 37.58 | 4.27 | <0.001 |
| Area | -33.46 | -43.67 | -23.24 | 5.21 | <0.001 |
| Mean longitude | -18.54 | -29.75 | -7.33 | 5.72 | 0.002 |
| Adjusted *R*^2^: 0.83 | | | | | |

Table S5. Beta regression of province-level inventory completeness for endemic species.

|  | Coefficient | Lower 95% CI | Upper 95% CI | Standard Error | *P*-value |
| --- | --- | --- | --- | --- | --- |
| Intercept | 1.25 | 0.82 | 1.69 | 0.22 | <0.001 |
| Human population density | 1.87 | 1.15 | 2.59 | 0.37 | <0.001 |
| Coast | -0.46 | -0.81 | -0.12 | 0.17 | <0.001 |
| Area | -1.55 | -2.53 | -0.56 | 0.50 | <0.001 |
| Mean latitude | 5.16 | 3.58 | 6.74 | 0.81 | <0.001 |
| Longitude:latitude | -5.04 | -6.48 | -3.61 | 0.73 | <0.001 |
| Pseudo *R*^2^: 0.74 | | | | | |

Figure S1. Moran’ I of (a) province-level mean discovery time and (b) residuals of the ordinary least-square regression.


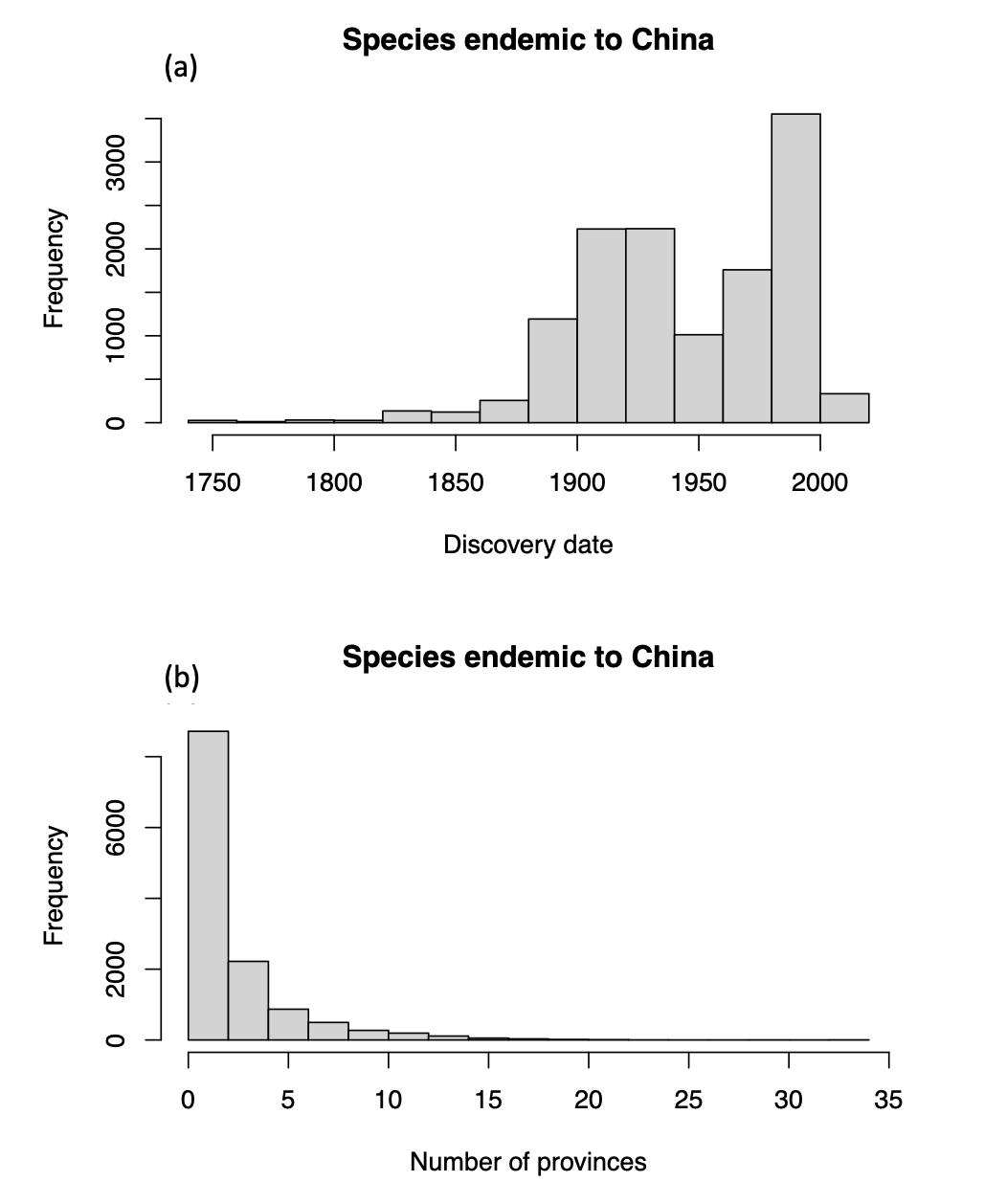


Figure S2. Histograms of discovery time (a) and the number of distributed provinces of endemic species to China (b).


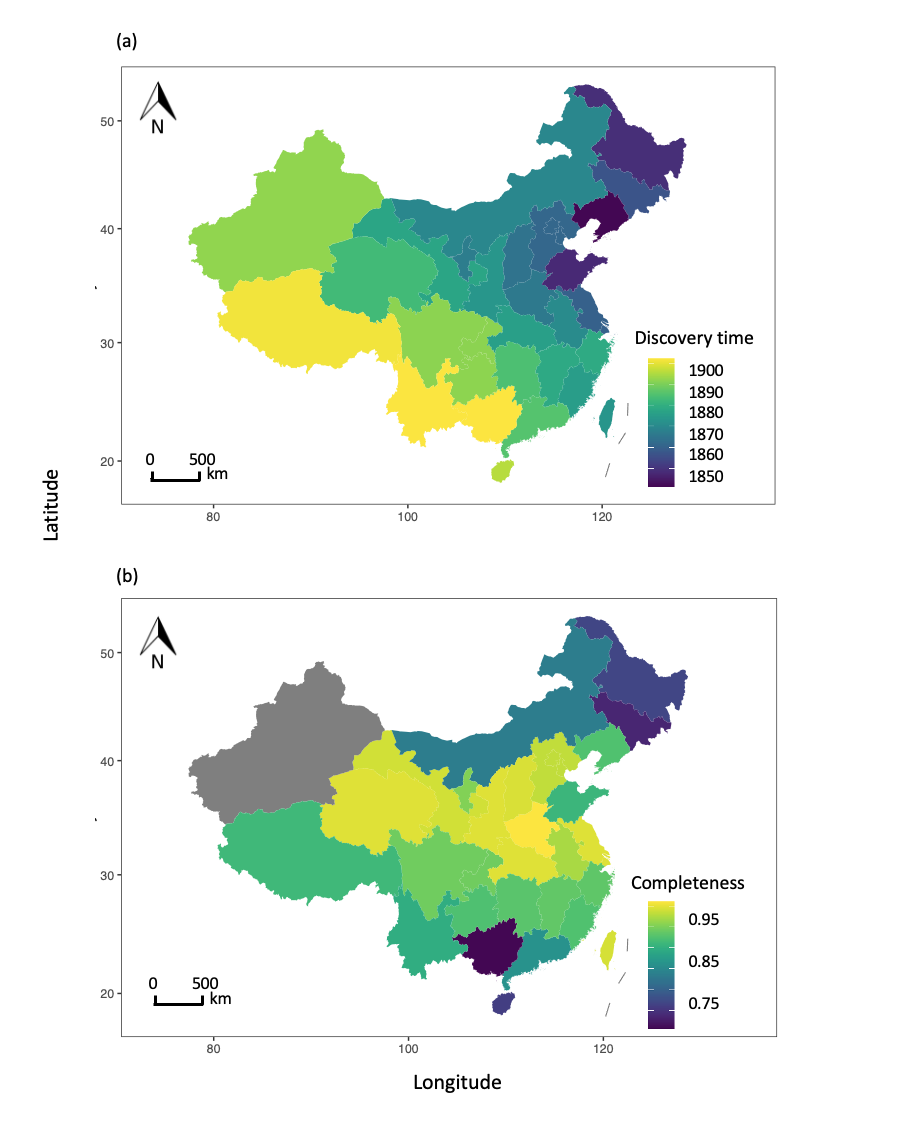


Figure S3. (a) Mean discovery time for province-level endemic species in China. (b) Species inventory completeness for province-level endemic species. Mollweide projection is used.


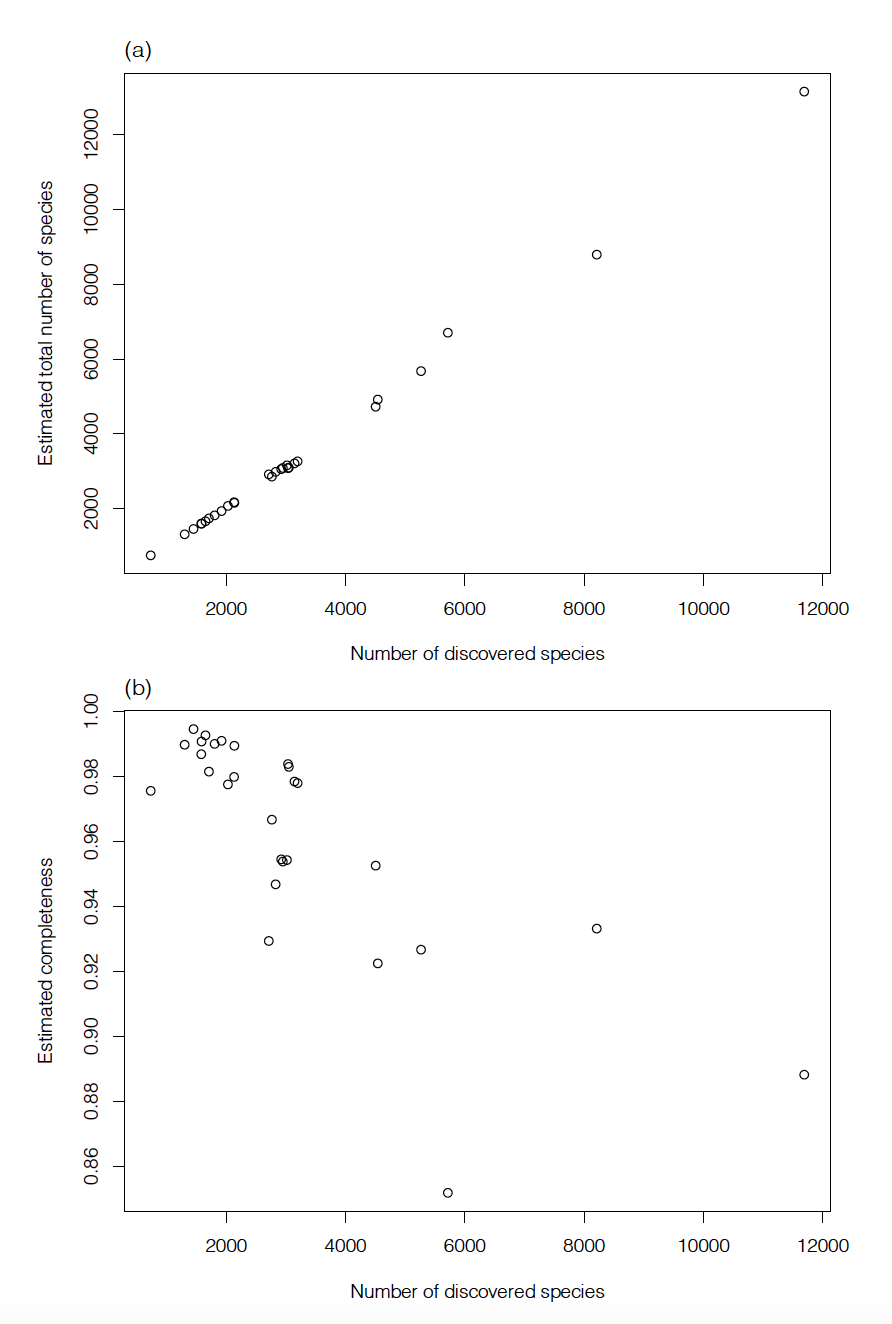


Figure S4. (a) Estimated total number of species against the number of discovered species at the province-level. (b) Estimated inventory completeness against the number of discovered species at the province-level.
